# Examining the weekend effect across ICU performance metrics

**DOI:** 10.1186/s13054-019-2479-5

**Published:** 2019-06-06

**Authors:** Louis Faust, Keith Feldman, Nitesh V. Chawla

**Affiliations:** Department of Computer Science & Engineering, University of Notre Dame, Notre Dame, USA

**Keywords:** Healthcare, Healthcare informatics, Weekend effect

## Abstract

**Background:**

Known colloquially as the “weekend effect,” the association between weekend admissions and increased mortality within hospital settings has become a highly contested topic over the last two decades. Drawing interest from practitioners and researchers alike, a sundry of works have emerged arguing for and against the presence of the effect across various patient cohorts. However, it has become evident that simply studying population characteristics is insufficient for understanding how the effect manifests. Rather, to truly understand the effect, investigations into its underlying factors must be considered. As such, the work presented in this manuscript serves to address this consideration by moving beyond identification of patient cohorts to examining the role of ICU performance.

**Methods:**

Employing a comprehensive, publicly available database of electronic medical records (EMR), we began by utilizing multiple logistic regression to identify and isolate a specific cohort in which the weekend effect was present. Next, we leveraged the highly detailed nature of the EMR to evaluate ICU performance using well-established ICU quality scorecards to assess differences in clinical factors among patients admitted to an ICU on the weekend versus weekday.

**Results:**

Our results demonstrate the weekend effect to be most prevalent among emergency surgery patients (OR 1.53; 95% CI 1.19, 1.96), specifically those diagnosed with circulatory diseases (*P*<.001). Differences between weekday and weekend admissions for this cohort included a variety of clinical factors such as ventilatory support and night-time discharges.

**Conclusions:**

This work reinforces the importance of accounting for differences in clinical factors as well as patient cohorts in studies investigating the weekend effect.

## Introduction

In 2001, a landmark study by Chaim Bell and Donald Redelmeier was released documenting an association between weekend admissions and patient mortality in hospital settings [1]. Understandably, the research quickly garnered a wealth of attention as the notion presented a highly undesirable, yet seemingly avoidable problem. However, over the two decades following the publication of Bell and Redelmeier’s work, a deluge of subsequent studies have emerged from the medical community further investigating what has become known as the “weekend effect” [2].

As the number of works investigating the weekend effect have continued to grow, an expansive body of literature has been generated associating the effect to a sundry of factors. Broadly, such factors have fallen into two primary schools of thought.

On one side, existing works have argued the effect manifests as a result of fundamental differences in the characteristics of patients admitted during the weekday and weekend: finding weekend patients to typically be of higher acuity than weekday patients. Coupling this with fewer admissions on the weekend overall, results in the seemingly inflated weekend mortality rates; leading many to relegate the effect to nothing more than an artifact of weekend census [3–6]. However, studies have found evidence of the effect even when adjusting for condition severity and within cohorts such as elective admissions; suggesting factors contributing to the effect extend beyond patient characteristics [7–10].

Beyond these characteristics, the other prevailing viewpoint focuses instead on aspects of caregivers themselves, specifically staffing, credentials, and seniority. From this perspective, prior work has shown patients receive less input from specialists, with hospitals staffing fewer specialists overall during the weekends [11]. Regarding general staff experience, studies suggest that teams are comprised of more junior doctors and nurses on weekend shifts [12]. In an effort to address such disparities in hospital staffing, the National Health Service in England adopted a 7-day service standard for emergency hospital care [13]. Yet, following a 3-year retrospective analysis, no significant changes in weekend mortality were associated with the implementation [14].

Despite the breadth of research into the phenomena, an understanding of the ways in which the weekend effect manifests remains elusive and often inconsistent [2, 15, 16]. In a prominent editorial published in *BMJ Quality and Safety*, Richard Lilford and Yen-Fu Chen suggest further work in replicating the weekend effect no longer serves a useful purpose [17]. Having established the weekend effect exists, the authors argue a shift in focus must be made to move beyond only identifying cohorts or circumstances in which it exists to more thorough investigations into what is causing it.

The work presented in this manuscript addresses such a call for advancement. Utilizing a large, publicly available, electronic medical record (EMR) database, we present a rigorous evaluation to isolate a cohort in which the weekend effect was most prevalent across intensive care settings. Then, utilizing the identified patient population, our work takes the next step: exploring the specific aspects of the effect’s manifestation. To do so, a novel analysis is presented to evaluate clinical factors across weekday and weekend admissions by employing a set of established measures of ICU performance.

Accordingly, the manuscript has been organized into two primary sections. First, we demonstrate the weekend effect to be most prevalent for surgical patients diagnosed with circulatory diseases. And second, we identify a set of statistically significant differences in ICU performance captured through a variety of well-established quality metrics such as ventilatory support and night-time discharges. By identifying these statistical differences, we motivate further investigation into the role of care in advancing our understanding of the weekend effect.

## Data and study population

### Data

The data utilized throughout the following manuscript were drawn from the “Medical Information Mart for Intensive Care” (MIMIC-III) repository. MIMIC-III represents one of the largest publicly available EMR databases, providing de-identified medical records for over 40,000 patients admitted to intensive care settings at the Beth Israel Deaconess Medical Center (BIDMC) in Boston, MA, USA, between 2001 and 2012 [18, 19]. As a level I trauma center, with 77 critical care beds (and a 620-bed tertiary academic medical center), BIDMC offers an excellent patient pool to explore the weekend effect across various patient cohorts. Moreover, as MIMIC-III provides a comprehensive overview of patient stays across multiple ICUs, including diagnoses, procedures, laboratory tests, prescriptions, and clinical notes, the data available present a unique opportunity to dig deeper into the manifestation of the effect across various care factors.

### Data preprocessing and cohort selection

**Fig. 1:**
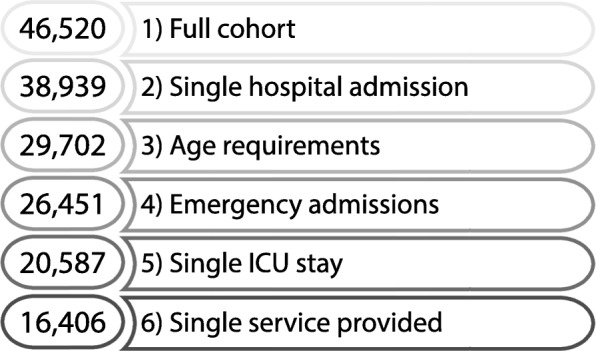
Outline of cohort selection

Although the MIMIC-III data is highly complete, prior to undertaking any analysis, we first performed a series of data preprocessing steps to address potential confounding factors. Specifically, focusing on those that arise from aspects of the data and how it was collected, external to the factors controlled for in our models. Each step is discussed in detail below and for the readers convenience, an outline of the criteria and the patient counts after each step is provided in Fig. 1.

Beginning with the full repository of 46,520 unique patients, we removed those with multiple (separate) hospital admissions (Fig. 1, level 2). This was done in order to protect against first, complications of a previous visit that may have exacerbated outside the clinical setting for which we have information and second, patients with an extremely high number of hospital readmissions (frequent flyers); leaving, in total, 38,983 patients.

Next, we focused on the de-identification process used across MIMIC-III. To comply with HIPAA regulations, all ages above 89 were masked with the value of “300” [18]. As this would preclude us from accurately controlling for age on this remaining population, we removed any such individual, leaving 37,345 patients. In a similar light, we also removed patients designated as *newborn*. Although newborns are recorded in the MIMIC-III database, their presence is the result of birth rather than illness and thus were removed. Note, only 41 infants were actually admitted to the neonatal ICU (NICU), supporting our decision to remove the newborn population. In total, these restrictions left 29,702 patients (Fig. 1, level 3).

From here, we focused on patients designated as elective admissions (Fig. 1, level 4). Since weekend admissions accounted for only 4% of elective patients (with weekday admissions accounting for 96%), this patient population was removed to help guard against the bias introduced by potential latent factors such as job status and transportation availability. These restrictions corresponded to a population of 26,448 patients remaining for analysis.

Within this population of patients, those admitted to multiple ICUs or readmitted to the same ICU were removed, resulting in 20,587 patients (Fig. 1, level 5). Such scenarios were omitted as multiple admissions at different points of the weekday could easily conflate the manifestation of a weekend effect. Finally, we removed patients receiving multiple types of care (deemed *services*) to isolate the service each patient received (Fig. 1, level 6). Overall, these criteria resulted in a robust study population of 16,406 unique patients. An overview of the demographics for this cohort are provided in Table 1. Additionally, we provide non-parametric statistical comparisons between the weekday and weekend groups, as well as a measure of effect size for all demographic values listed.

**Table 1:** Overview of cohort demographics

	Week, *N*(%)	Weekend, *N* (%)	*P* (effect size)
Age			
	62 ± 18 years	59 ± 20 years	<.001 (0.15)
Sex			
Male	6863 (57)	2485 (57)	0.76 (0.002)
Female	5197 (43)	1861 (43)	
Ethnicity			
White	8264 (68)	2881 (66)	0.01 (0.03)
Black/African American	961 (8)	335 (8)	
Hispanic/Latino	434 (4)	169 (4)	
Asian	284 (2)	104 (2)	
Other	335 (3)	159 (4)	
Unknown	1782 (15)	698 (16)	
Time of day			
12:00 AM - 7:59 AM	2375 (19)	1172 (27)	<.001 (0.08)
8:00 AM - 5:59 PM	5015 (42)	1624 (37)	
6:00 PM - 11:59 PM	4670 (39)	1550 (36)	
Direct ICU admit?			
Yes	8668 (72)	3298 (76)	<.001 (0.04)
No	3392 (28)	1048 (24)	
Diagnosis			
Diseases of the circulatory system	4287 (36)	1205 (28)	<.001 (0.10)
Injury and poisoning	2543 (21)	1184 (27)	
Diseases of the digestive system	1155 (10)	468 (11)	
Infectious and parasitic diseases	968 (8)	416 (10)	
Diseases of the respiratory system	1044 (9)	376 (9)	
Endocrine/nutritional diseases*	358 (3)	144 (3)	
Neoplasms	532 (4)	137 (3)	
Mental Illness	299 (2)	137 (3)	
Diseases of the nervous system*	313 (3)	111 (2)	
Genitourinary system diseases	228 (2)	75 (2)	
Ill-defined conditions*	79 (< 1)	29 (< 1)	
Diseases of the blood*	46 (< 1)	18 (< 1)	
Musculoskeletal system diseases*	84 (< 1)	17 (< 1)	
Diseases of the skin*	40 (< 1)	10 (< 1)	
Complications of pregnancy*	30 (< 1)	10 (< 1)	
Congenital anomalies	41 (< 1)	8 (< 1)	
Residual codes	13 (< 1)	1 (< 1)	
OASIS severity score (min, med, max)			
	(0.65, 9.78, 92.38)	(0.65, 9.78, 94.0)	0.49 (0.01)
Service			
Medical	7896 (65)	2897 (67)	<.001 (0.09)
Surgical	2875 (24)	766 (18)	
Trauma	1160 (10)	630 (14)	
Other	129 (1)	53 (1)	

*Label has been truncated; full text is available in the CCS manual [22]

## Methods

In line with the two overarching questions of this manuscript, the analyses are broken into two distinct groupings: first, multiple logistic regression was utilized to identify and isolate a specific cohort in which the weekend effect manifests and second, exploring statistical differences in ICU performance through a set of established clinical factors. Details of each can be found in the corresponding sections to follow.

### Part 1—Identifying a patient cohort

The first step in our analysis intended to address whether the weekend effect was pervasive across the set of 16,406 patients or if it occurred primarily in one or more specific cohorts.

#### Model specification

Logistic regression was utilized to allow us to investigate the relation of specific factors to a discrete outcome (in this case mortality), while adjusting for a number of known confounding variables. In particular, we focused on three primary sets of confounding factors: patient demographics, admission characteristics, and diagnostic characteristics.

With respect to patient demographics, we controlled for age, sex (*male*, *female*), and ethnicity (*white*, *black/African American*, *Hispanic/Latino*, *Asian*, *other*, *unknown*).

For admission characteristics, we adjust for time of day, discretized into three periods: day (8:00 AM to 5:59 PM), evening (6:00 PM to 11:59 PM), and night (12:00 AM to 7:59 AM) [20]. Although all patients considered were listed as emergency admits, we further control for their location prior to admit: creating a binary feature to separate patients already in a hospital bed from those directly admitted to the ICU.

Regarding diagnostic characteristics, we included patients’ primary diagnosis to help account for the mortality risk of various conditions. Rather than using the breadth of discrete ICD-9 CM codes, we instead utilized the Clinical Classifications Software (CCS) clinical grouper, a tool for clustering patient diagnoses and procedures into a manageable number of clinically meaningful categories [21]. Specifically, we utilize the mapping from five-digit ICD-9 CM codes into the 16 level 1 categories provided by CCS [21, 22]. Further, we controlled for condition severity, measured by the Oxford Acute Severity of Illness Score (OASIS) [23, 24].

Finally, starting at the highest level of care designation available in MIMIC-III, we included the factor of *service*, which represented the type of care the patient was admitted under, separated into *medical*, *surgical*, *trauma*, and *other*. An interaction was placed between weekend and service type in the logistic regression model to capture variations in mortality rates across the different services for weekday and weekend admissions. Further, we note two design choices made in constructing this model. First, we define weekend as Saturday morning 00:00:00 (12 AM) through Sunday evening at 23:59:59 (11:59 PM). Second, mortality represents any in-hospital mortality, i.e., patient death prior to discharge from the respective hospital admission.

#### Post hoc analysis

Identifying the weekend effect with our logistic model, we then sought to determine whether a more granular cohort existed within this population where the weekend effect was most prevalent. To do so, patients within the cohort were stratified by their primary diagnoses (CCS code Level-1), mortality rates among weekday and weekend patients were then compared utilizing Fisher’s exact tests.

### Part 2—ICU performance metrics

Building on the observations from part 1, we turned to novel analyses exploring the manifestation of the weekend effect with respect to ICU performance across various clinical factors. However, directly comparing aspects of care across patients provides an unstable and often unreliable assessment of differences, particularly when compared to a single outcome such as mortality.

As a result, we look instead to a field that has been a long-standing focus among the medical community: the evaluation of the quality and effectiveness of care within a hospital or unit. While the notion of care is an arbitrary concept, the need for effective measures has led to the development of well established clinical *scorecards*. These scorecards represent collections of performance metrics widely agreed upon by medical professionals as a reflection of care quality. On one side, they offer a standardized manner in which to evaluate performance on the individual level i.e. nurses and physicians, as well as the group level: emergency rooms or ICUs. While in another light, they are not unit specific and can be applied to research across various services and conditions.

Focused on the specific patient cohort identified within part 1 of this analysis, we utilized a set of established performance metrics aggregated from two clinical scorecards focused on intensive care settings. First, the Ontario Critical Care Unit Balanced Scorecard, which was selected from the World Health Organization’s *Catalog of resources to support health services delivery transformations* for its focus on examining critical care [25, 26]. Second, we utilized the Critical Care Medicine-specific Quality Scorecard. Drafted in 2018 and derived from evidence-based, validated tools, the scorecard is focused on intensive care performance measurement [27].

It should be noted that because these scorecards were not developed for the de-identified data used in this study, MIMIC-III lacks the data necessary to derive all of the proposed metrics. Nonetheless, the best effort was made to compute as many as possible, resulting in a final set of 12 metrics capturing a wide array of care factors. These factors can be found in Table 4, while a full listing of performance metrics can be found in each of the respective scorecards [25, 27]. As we made no assumptions regarding underlying distributions, non-parametric Fisher’s exact and Mann-Whitney *U* tests were computed where appropriate to test for statistical differences between weekday and weekend cohorts.

Finally, we sought to ensure any identified differences in performance were reflective of the weekend effect rather than a product of broader differences across weekend ICU operations. Therefore, the scorecard analyses were also computed among the remaining service cohorts in which no significant weekend effect was prominent: medical, trauma, and other.

## Results

In an effort to provide consistency, the results of our analyses are organized into two primary sections aligned with the methods detailed in the “Methods” section.

### Part 1—Identifying a patient cohort

**Fig. 2:**
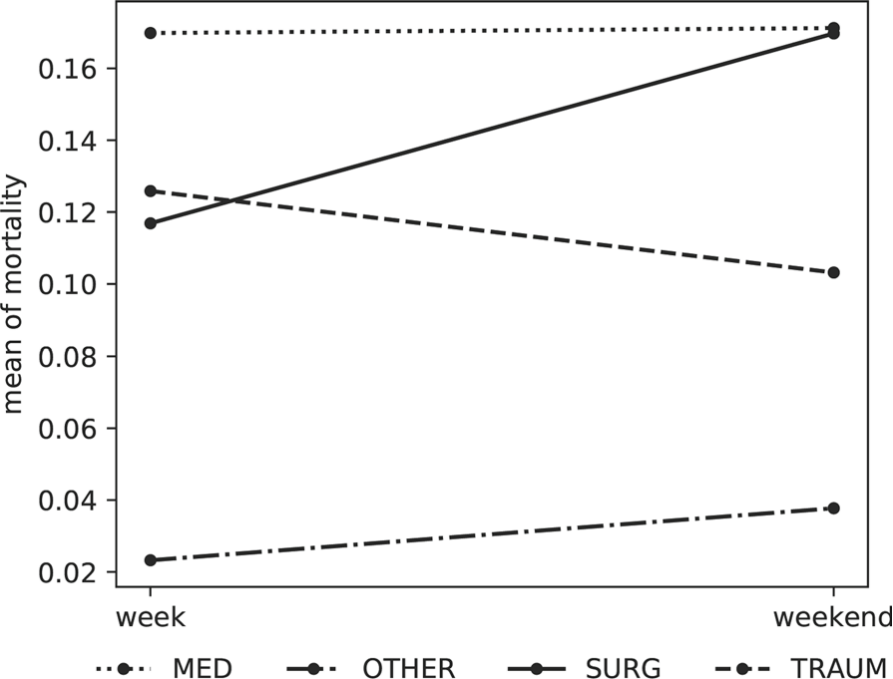
Odds plot of weekday and weekend admission mortality stratified by service type

Looking first to the identification of a patient cohort in which the weekend effect can be most clearly observed, we turn to the results of the logistic model, found in Table 2. Our interest falls to the interaction between weekend and service type. Here, we find that mortality among patients admitted to an ICU on the weekend for *surgical* treatment is significantly higher than weekday ICU admissions (aOR 2.03; 95% CI 1.52, 2.71). An interaction plot illustrating this relation is provided in Fig. 2. Other services including *medical*, *trauma*, and *other* remain fairly constant across patients admitted on the weekday and weekend.

**Table 2:** Results from the logistic regression model interaction term between weekend and service

	Unadjusted^†^ (*n* = 16,406)	Adjusted^‡^ (*n* = 16,406)
Weekend mortality by service*	OR (95% CI)	aOR (95% CI)
Medical	Reference	Reference
Surgical	1.53 (1.19, 1.96)	2.03 (1.52, 2.71)
Trauma	0.79 (0.57, 1.10)	0.95 (0.65, 1.38)
Other	1.63 (0.26, 10.09)	1.10 (0.14, 8.75)

*Weekday is the reference category for this interaction

^†^Includes only the interaction between weekend and service

^‡^Adjusts for sex, age, ethnicity, time of day, prior hospital admission, diagnosis, and condition severity

It is important to note that the medical service was utilized as the reference category in our logistic regression as it is recommended to use the largest group when no specific group comparisons are of interest [28]. However, to ensure the generalizability of our observation, the significance among surgical patients was also tested with reference categories of *trauma* and *other*, remaining significant at *P*<.05 in all cases.

#### Post hoc analysis

Building off the results of the logistic model, we identified a more granular cohort in which the result was most prevalent. Referring to Table 3, we find *Diseases of the circulatory system* exhibits the greatest disparity between mortality rates (15%), with a significantly higher rate present for weekend admissions (*P*<.001). As no other significant differences in mortality rates were present among the other more common diagnoses, we move to part 2, utilizing the cohort of 1708 surgical patients with a primary diagnosis category of *Diseases of the circulatory system*.

**Table 3:** Weekday and weekend mortality rates across the top five most prevalent diseases for surgical patients in the cohort

CCS level 1 label	Weekday population	Weekend population	Weekday mortality rate (%)	Weekend mortality rate (%)	*P*
Diseases of the circulatory system	1449	259	12	27	<.001
Injury and poisoning	586	250	12	12	1.00
Diseases of the digestive system	302	118	7	8	0.67
Neoplasms	246	50	7	4	0.54
Infectious and parasitic diseases	72	34	35	32	1.00

**Table 4:** Overview of differences in ICU performance metrics between weekday and weekend patients

Care metric	Week, *n* = 1449	Weekend, *n*= 259	Effect size (*P*)	Scorecard
Deliver safe care				
VAP rate	1%	3%	4.16^OR^ (.004)	OCCUBS
CLI rate	0%	0%	NA	OCCUBS
Optimize patient flow				
ICU length of stay	4.48 days	6.72 days	0.35^CD^ (<.001)	OCCUBS
Access				
Admission to (ICU) bed, > 90 min	51%	65%	1.82^OR^ (<.001)	OCCUBS
Night-time discharge rate (10:00 PM–7:00 AM)	10%	16%	1.83^OR^ (.002)	OCCUBS
Ventilation and weaning				
Unplanned extubation	0.3%	0.8%	2.81^OR^ (.23)	OCCUBS
% of patients maintained on a ventilator	78%	60%	0.41^OR^ (<.001)	CCMQS
Reintubation rate, < 48 h after planned extubation	11%	19%	2.08^OR^ (.002)	CCMQS
Fluid balance				
Median fluid balance	2.96 L	3.27 L	0.21^CD^ (.03)	CCMQS
Median fluid balance/24 h	0.18 L	0.14 L	0.09^CD^ (.03)	CCMQS
Prevention				
% of patient days with blood glucose >120 mg/dL	85%	83%	0.06^CD^ (.38)	OCCUBS
% patient days with central line	49%	48%	0.01^CD^ (.008)	OCCUBS

*OCCUBS* Ontario Critical Care Unit Balanced Scorecard, *CCMQS* Critical Care Medicine-specific Quality Scorecard, *VAP* ventilator-acquired pneumonia, *CLI* central line infection, *OR* odds ratio, *CD* Cohen’s *d*

### Part 2—ICU performance metrics

Drawing on the patient cohort identified in part 1, our analyses in the following section highlights how established performance metrics offer insight into the manifestation of the weekend effect. Table 4 presents an overview of the 12 derived scorecard metrics stratified by weekday and weekend admissions, also detailing which scorecard the metric was drawn from. These metrics provided a number of differences from both care elements and logistical factors for patients across weekend and weekday admissions.

From a logistical perspective, the prevalence of visits where the length of time between hospital arrival and admission to an ICU was greater than 90 min was higher for weekend admissions (OR 1.82). Additionally, the average ICU stay was 1 day longer for a weekend admission than a weekday admission (*d* 0.35), and the prevalence of night-time discharges was also higher for weekend admissions (OR 1.83).

With respect to more care-focused metrics, we note a smaller percentage of patients maintained on a ventilator was observed for the weekend (OR 0.41). However, a higher rate of ventilator acquired pneumonia and an elevated reintubation rate after a planned extubation were observed on the weekend as opposed to the weekday (OR 4.16, OR 2.81 respectively). For fluid balance, a higher median fluid balance was observed for weekend patients across their entire stay (*d* 0.21). Additionally, for prevention metrics, weekday admitted patients typically had a central line for a longer portion of their stay than weekend admitted patients (*d* 0.01). It should be noted that, within the cohort studied, only one instance of a central line infection was recorded, as such, differences could not be defined.

Having repeated our scorecard analyses across the remaining service cohorts, referring to Table 5, we observed significant differences within the *other* cohort as well. Such differences are in line with the observations in Fig. 2, given *other* exhibits a slight increase between weekday and weekend mortality. However, as *other* represents the aggregation of many disparate (small *n*) services, all of which likely do not exhibit the effect, the association was not strong enough to be statistically significant in the initial analysis.

**Table 5:** Overview of differences in ICU performance metrics across service cohorts

Care metric	Trauma	Other	Medical	Surgery, circulatory
Deliver safe care				
VAP rate^OR^	0.91 (1.0)	NA	1.41 (.06)	*4.16 (.004)*
CLI rate^OR^	NA	NA	1.81 (.47)	NA
Optimize patient flow				
ICU length of stay^CD^	0.11 (.19)	0.002 (.15)	0.04 (.01)	*0.35 (<.001)*
Access				
Admission to (ICU) bed^OR^	0.79 (.04)	INF (<.001)*	1.18 (<.001)	*1.82 (<.001)*
Night-time discharge rate^OR^	0.75 (.06)	0.49 (.40)	1.06 (.34)	*1.83 (.002)*
Ventilation and weaning				
Unplanned extubation^OR^	0.81 (1.0)	NA	0.87 (.77)	2.81 (.23)
% of patients maintained on a ventilator^OR^	1.20 (.07)	*0.42 (.01)*	1.08 (.08)	*0.41 (<.001)*
Reintubation rate, < 48 h after planned extubation^OR^	0.71 (.05)	0.80 (1.0)	0.98 (.88)	*2.08 (.002)*
Fluid balance				
Median fluid balance^CD^	0.05 (.05)	0.30 (.09)	0.01 (.003)	*0.21 (.03)*
Median fluid balance/24 h^CD^	0.09 (<.001)	*0.40 (.006)*	0.02 (<.001))	0.09 (.03)
Prevention				
% of patient days with blood glucose > 12^CD^	0.17 (.02)	0.09 (.32)	0.03 (.14)	0.06 (.38)
% patient days with central line^CD^	0.16 (.08)	0.02 (.26)	0.01 (.17)	0.01 (.008)

Results are presented as effect sizes (*P* values)

OR odds ratio, CD Cohen’s *d*, *NA* not available

*No weekend patients observed with admissions to ICU bed under 90 min

## Discussion

As questions surrounding the relationship between weekend admission and mortality continue to circulate, the analyses presented within this manuscript provide an opportunity to understand the patient populations most at risk and the value in examining the role of ICU performance metrics. In particular, the following section reflects on several observations that advance our understanding of the weekend effect.

We begin with insights into the weekend effect found at the highest level of our patient cohort (Fig. 1, level 6). From our regression analysis, we find that rather than a pervasive increase in mortality, the effect was instead localized to specific cohorts. By allowing mortality probabilities to vary across the type of services patients received (Fig. 2), we observed a 4% increase in mortality for surgical patients. Investigating these surgical patients further, we find this increase was driven primarily by patients diagnosed with *Diseases of the circulatory system*.

In line with the overarching effort of this work to advance knowledge of the weekend effect, having identified a cohort in which the effect was most prevalent, we now turn to the ways in which it manifests within a population. Specifically, those performance metrics found to significantly differ between weekday and weekend admissions.

Looking first to metrics which closely pertain to clinical elements, we find several related to ventilatory care. Overall, ventilator-associated pneumonia (VAP) incidents were higher among patients admitted during the weekends. While attributable VAP mortality has been shown to be near zero in trauma and medical patients, for surgical patients, attributable VAP mortality has been shown to be as high as 13% [29].

This finding is particularly interesting when viewed in the broader context of the scorecard metrics. We find the percentage of patients maintained on a vent was lower for patients admitted on the weekend; however, their reintubation rate (within 48 h of a planned extubation) was higher. Given reintubation has been shown to increase risk of VAP and prior work has shown clinically significant procedural complications become more common in repeated intubation, these observations clearly exhibit the value in the utilization of clinical factors and performance metrics in broadening our understanding of the weekend effect [30, 31].

Moving to logistical metrics, we find similar insights when comparing the outcomes of weekday and weekend patient admissions. We observed increased rates of night-time discharges, defined as occurring between the hours of 10:00 PM and 7:00 AM across the total population of patients admitted on the weekend. Night-time discharges have been associated with increased mortality risk as premature discharges have been shown to become more common within these hours [32, 33]. Further, patients admitted during the weekends had, on average, longer ICU stays by 24 h. While a variety of factors can influence length of stay such as age and severity of illness, patients with longer ICU stays are prone to higher risk of ICU-acquired infections [34, 35]. Finally, we observed that among those directly admitted to the ICU, a higher percentage of weekend patients were in the emergency room for longer than 90 min before being admitted to an ICU bed, a significant factor for critically ill patients [36].

While it is important to acknowledge these factors are not able to provide a causal link between weekend admission and mortality rates within an intensive care environment, they shed new light on ways in which the weekend effect may manifest. Moreover, we believe the methodology of studying ICU performance metrics and clinical factors offers an important foundation for the continued advancement of research surrounding the weekend effect, and ultimately the ways in which such effects could be addressed.

## Limitations

Although the utmost consideration was given to the technical correctness and evaluation methodologies performed in the analyses throughout this manuscript, there are two primary limitations to the study which must be discussed.

First, given the MIMIC-III dataset includes records from 2001 to 2012, evidence of the weekend effect and differences in care may be biased by earlier records which no longer reflect today’s current clinical practices as studies have shown reduced morality for weekday and weekend admissions over time [37]. In order to protect patients anonymity, all dates stamps were masked, preventing us from discerning whether this bias exists.

Second, not all scorecard metrics were readily computable using the MIMIC-III database, leaving only a limited range of ICU performance metrics that could be analyzed.

Further, given the MIMIC-III dataset is constrained to records from one hospital and sample size is limited, additional studies are necessary across different populations and environments to validate these findings.

## Conclusion

Although a wealth of studies have investigated the weekend effect, limited work has moved beyond identifying the effect to understanding how it manifests. This work addresses this deficiency: establishing the relation of care factors to weekend ICU mortality. Such findings establish the value in employing a more comprehensive view of clinical settings and present clear elements that can act as the basis for improvement programs moving forward.
